# Automated Real-Time PCR Detection of Tickborne Diseases Using the Panther Fusion Open Access System

**DOI:** 10.1128/spectrum.02808-22

**Published:** 2022-11-14

**Authors:** Kathleen A. Stellrecht, Lisa I. Wilson, Allan J. Castro, Vincente P. Maceira

**Affiliations:** a Department of Pathology and Laboratory Medicine, Albany Medical Center Hospital, Albany, New York, USA; b Department of Pathology and Laboratory Medicine, Albany Medical College, Albany, New York, USA; c Department of Immunology and Microbial Diseases, Albany Medical College, Albany, New York, USA; Quest Diagnostics

**Keywords:** tickborne diseases, *Anaplasma phagocytophilum*, *Ehrlichia chaffeensis*, *Babesia microti*, multiplex real-time PCR, Panther Fusion, Open Access, lab developed tests (LDT), automation

## Abstract

The incidence of tickborne infections in the United States has risen significantly. Automation is needed for the increasing demand for testing. The Panther Fusion (Fusion) has an Open Access functionality to perform lab developed tests (LDTs) on a fully automated system. Our laboratory adapted two LDTs on Fusion; a multiplex real-time PCR for Anaplasma phagocytophilum and Ehrlichia chaffeensis (AP/EC) and a Babesia microti (BM) PCR. Limits of detection (LODs) were performed with target region plasmid panels spiked into whole blood. The LODs for AP, BM, and EC on the Fusion were 11, 17, and 10 copies/reaction, respectively. The performance of AP/EC was evaluated with 80 whole blood specimens, including 50 specimens previously positive for AP by our test of record (TOR) and 30 specimens (including 20 AP positive) spiked with EC plasmid. AP was detected in 49 out of 50 positive specimens and EC was detected in all 30 spiked specimens. BM PCR on Fusion was evaluated with 75 whole blood samples, including 16 specimens previously shown to be positive for BM and 59 negative specimens, of which 29 were spiked with BM plasmid DNA. BM was detected in 45 samples as expected. AP/EC and BM PCRs were successfully developed and optimized on the Panther Fusion with performance characteristics comparable to our TOR. These assays complement each other and allow for a modular testing approach for tickborne diseases which have differing clinical presentation. Furthermore, automation of these assays will help the lab meet the increasing demand for testing.

**IMPORTANCE** Since the incidence of tickborne diseases has been accelerating in the United States, automation for testing has become essential in affected regions. Unfortunately, because the need is regional, commercial test manufacturers have not yet provided answers for clinical laboratories. Here, we describe the development of PCR tests on the highly automated Panther Fusion for three tickborne diseases. The Panther Fusion assays were evaluated using 155 archived whole blood (WB) specimens previously tested for Anaplasma phagocytophilum, Ehrlichia chaffeensis, and Babesia microti, while WB spiked with DNA from plasmid clones of the target regions were used for analytical sensitivity. We demonstrated that the Panther Fusion assays performed similar to the manual PCR tests used clinically in our laboratory and that automation of these tests had no adverse effect on the performance.

## INTRODUCTION

Anaplasma phagocytophilum (AP) and Ehrlichia chaffeensis (EC) are Gram-negative obligate intracellular bacteria that reside and propagate within membrane-lined vacuoles found in the cytoplasm of granulocytes and monocytes, respectively, and are the causative agents of human granulocytic anaplasmosis (HGA) and human monocytic ehrlichiosis (HME). Babesia microti (BM) is an erythrocytic parasite that is the primary cause of babesiosis in the United States. The commonality of these diseases are they are all tickborne diseases. AP and BM are most commonly transmitted by the blacklegged tick, Ixodes scapularis. The geographic range of this tick has been expanding across New York State (NYS), and over the past decade, the NYS Capital District region has been experiencing a surge in I. scapularis-borne diseases (1, 2). The primary vector for EC is *Amblyomma americanum*, the lone star tick. Although this tick has its highest population densities in the south-central and southeastern United States, it too is spreading geographically and increasingly EC infections are being identified in both the mid-Atlantic and northeastern regions (https://www.cdc.gov/ticks/tickbornediseases/index.html) (3).

HGA and HME often begin with a prodrome characterized by malaise, low-back pain, or gastrointestinal symptoms that may be followed by sudden onset of fever, headache, myalgias, arthralgias, and malaise (3). Disease presentation can range from mild to life-threatening with multiorgan system involvement. Other manifestations include respiratory symptoms (cough, pharyngitis), lymphadenopathy, rash, and even changes in mental status. Babesia infection can also range from asymptomatic, to nonspecific influenza-like disease with malaise followed by fever with chills or sweats, to life-threatening complications with multiorgan system involvement, hemolytic anemia, and thrombocytopenia (4).

Diagnosis begins with clinical and epidemiological factors. Patients presenting with fever, leukopenia, thrombocytopenia, elevated serum aminotransferase levels, and a history of recent tick bite in regions of endemicity should be considered possibly having HGA or HME, particularly from May through July, and again with the start of NYS’ deer hunting season in November, when most infections occur. Morulae are observed in 20% to 80% of patients with HGA but less than 7% of patients with HME (5–7). Babesiosis should be considered in patients with history of tick bites who present with fever with chills or sweats. Parasites are seen in Giemsa-stained thin blood smears; however, when parasitemia is low, sensitivity is lacking (8).

Cultures for AP and EC are not routinely available nor are they clinically useful because they require prolonged incubation times (more than a week) (9, 10). Serology is only useful, retrospectively, with 22% to 44% of cases having a significant titer in the acute phase of illness. Furthermore, 15% to 16% of the population in the upper Midwest and New York State are seropositive (11, 12); hence, convalescent-phase sera with a 4-fold rise in titer is necessary for diagnosis. Nucleic acid amplification tests such as PCR are recommended for AP and EC diagnostic testing during acute infection because of the higher sensitivity and clinically meaningful turnaround times.

Our laboratory adapted both a multiplex real-time PCR targeting the AP *msp2* and the EC *dsb* genes (13) and a separate assay targeting BM specific 18S rRNA gene sequences (14). We began clinical testing for these pathogens in 2016 and since then, the testing volume has increased dramatically as the geographic range of tickborne diseases has expanded through eastern NYS. Hence, automation is vital to address the need for more testing. The Panther Fusion is a fully automated system integrating nucleic acid extraction, with either transcription mediated amplification (TMA) or real-time PCR. Fusion also has Open Access functionality for PCR based LDTs with full automation. Our laboratory has adapted our tickborne disease PCRs on the Fusion and we are reporting the evaluation of these two LDTs.

## RESULTS

DNA from the three target plasmids were used to create 3 member panels, prepared as 10-fold serial dilutions in WB, for the optimization experiments. Optimization studies began with optimal primer concentration analyses, followed by probe titrations using initial reagent concentrations of 8 mM Tris, 4 mM MgCl_2_, 50 mM KCl, based on experience with LDT assay development on the Fusion. For the multiplex assay, primers and probes of individual targets were analyzed as uniplex assays and then combined into the multiplex prior to reagent titration. Table 1 lists the optimal concentrations for the primer and probes. For reagent titration Tris was optimized first followed by KCl and then MgCl_2_ and the optimal reagents concentration are detailed in Table 2.

**TABLE 1:** Primers and probes^*a*^

		Concn (μM) per reaction	
ID	Oligonucleotide sequence (5′–3′)	TOR	Fusion
Primers			
ApMsp F	GTA-GCT-ATG-GAA-GGT-AGT-GTT-GGT-TAT-G	0.7	0.55
ApMsp R	TTG-GCT-TTG-AAG-CGC-TCG-TA	0.7	0.55
EcDsb F	TTG-GAG-AAG-CAT-CAC-TGA-AAG-C	0.7	0.55
EcDsb R	GCA-GCA-TGG-TAG-AAC-TCG-ATG-TA	0.7	0.55
Babesia 8 F	CAG-GGA-GGT-AGT-GAC-AAG-AAA-TAA-CA	0.1	0.4
Babesia 8 R	GGT-TTA-GAT-TCC-CAT-CAT-TCC-AAT	0.1	0.4
UIC DNA F& R	Proprietary	NA	1×
Probes			
ApMsp2 Pr	FAM-TGG-TGC-CAG-GGT-TGA-GCT-TGA-GAT-TG/BHQ	0.2	0.4
EcDsb Pr	TexRd-XN/TCA-AGC-AGC-ACT-AGC-AGT-ACA-TCT-AAT-AAA-CCC-AAG TAA/BHQ_2	0.3	0.6
B. microti 8	FAM-TAC-AGG-GCT-TAA-AGT-CT-MGBNFQ	0.1	0.5
Bicoid Pr	VIC-TCG-CTC-TGT-TTC-ATA-CCC-GGC-GA-TAMRA	0.2	NA
BBIC	VIC-CTA-CAA-CTT-CAA-CAG-CTC-GTA –MGBNFQ	0.05	NA
UIC DNA Pr	Proprietary	NA	1×

*^a^*NA, not applicable.

**TABLE 2:** Primer probe reconstitution (PPR) mix reagent concentrations

	Concn (mM)	
Reagent	AP/EC	BM
Tris	8	10
MgCl_2_	6	8
KCl	75	75

**FIG 1:**
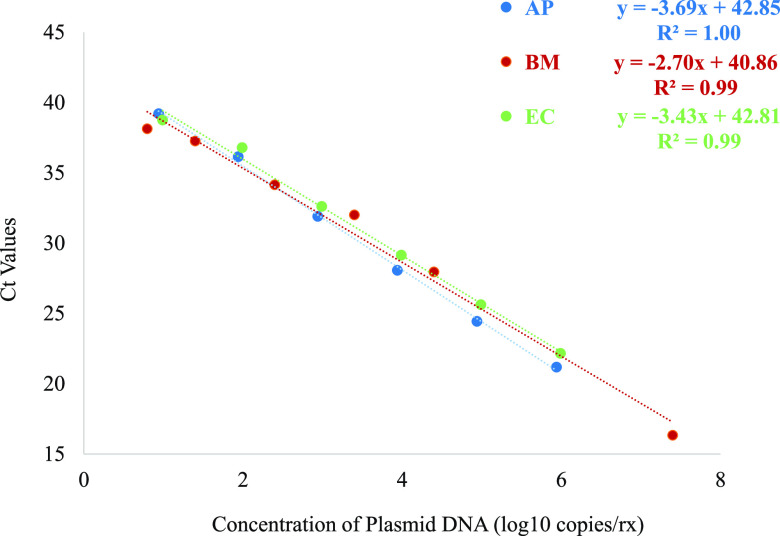
Linear regression analysis of AP, BM and EC targets on the Panther Fusion with dilution panels. Each dilution of plasmid DNA spiked into WB was tested 4 to 10 times and the mean Ct value at each concentration were plotted. The formulas represent the linear regression equation along with the correlation coefficients for each LDT.

DNA from the three target specific plasmids were also used to create a series dilution and tested four to 10 times for LOD and precision analysis. From these studies, the LODs for AP, BM and EC were 11, 17 and 10 copies/reaction, respectively, on the Fusion (Table 3). Historical LOD data for TOR were 11, 15, and 11, respectively, for the three targets. The coefficient of variance (CV) for the intraassay and interassay Ct values, across three concentrations of DNA, ranged from 0.5% to 2.1% for AP, 0.3% to 2.6% for EC, and 1.0% to 4.9% for BM. These studies also demonstrated the Ct values correlated linearly with expected efficiencies and with R2 ranging from 0.99 to 1.0 (Fig. 1).

**TABLE 3:** Limit of detection analyses

Concn (log copies/rx)	N	Rate	Mean Ct	Std	CV (%)
Anaplasma phagocytophilum *(LOD = 1.02 Log copies/rx)*					
5.94	7	1	21.19	0.27	1.3
4.94	6	1	24.45	0.52	2.1
3.94	7	1	28.07	0.35	1.3
2.94	6	1	31.92	0.78	2.4
1.94	7	1	36.16	0.55	1.5
0.94	10	0.7	39.21	0.94	2.4
0.34	4	0	–^*a*^	–	–
−0.06	7	0	–	–	–
Babesia microti *(LOD = 1.23 Log copies/rx)*					
7.40	4	1	16.34	0.83	5.1
4.40	7	1	27.96	1.19	4.3
3.40	8	1	32.01	0.78	2.4
2.40	8	1	34.16	1.56	4.4
1.40	8	1	37.27	0.74	2.0
0.80	7	0.71	38.14	0.42	1.1
0.40	8	0.25	40.20	1.70	4.2
−0.60	7	0	–	–	–
Ehrlichia chaffeensis *(LOD = 1.00 Log copies/rx)*					
5.99	7	1	22.17	0.53	2.4
4.99	6	1	25.63	0.48	1.9
3.99	7	1	29.16	0.75	2.6
2.99	6	1	32.62	0.54	1.7
1.99	7	1	36.80	0.90	2.4
0.99	10	0.9	38.77	1.24	3.2
0.39	4	1	38.98	0.78	2.4
−0.01	7	0	–	–	–

*^a^*The dashes indicates for dilutions that had no positive samples hence no statistical information.

**FIG 2:**
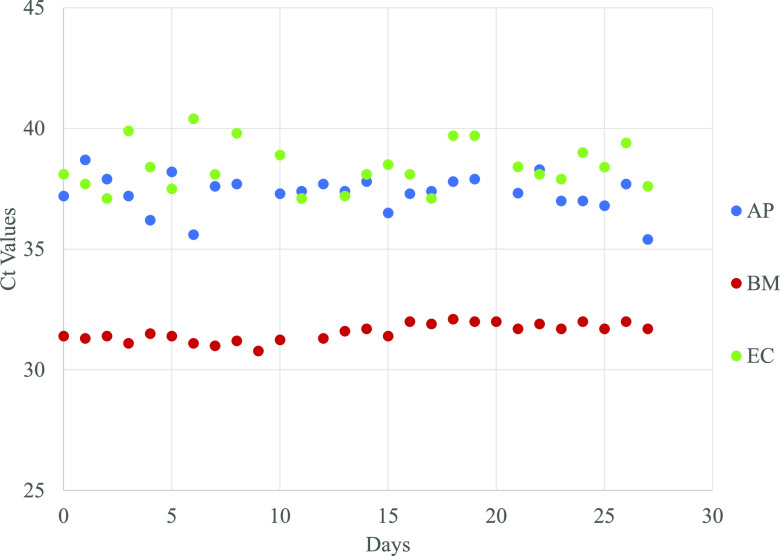
Onboard stability of the PPR mixes. CT values over a period of 27 days for plasmid DNA spiked into BTM treated WB tested.

To test the stability of the PPR mixes, the mixes were left onboard the instrument at room temperature and the positive controls were tested almost daily for 27 days. The CT values remained stable for the entire test period (mean ± SD = 37.32 ± 0.66 for AP; 31.59 ± 0.36 for BM; 38.41 ± 0.97 for EC) and all replicates were detected (Fig. 2). These results demonstrated the onboard stability of the PPR mix for at least 27 days.

A panel of 15 vector-borne pathogen DNA, provided by the NYS Wadsworth Center, were spiked into whole blood for analytical specificity studies. These pathogens included AP, EC, BM, *Babesia odocoilei*, Bartonella henselae, Borrelia burgdorferi *B31*, Leishmania amazonensis, *Leishmania guyanensis*, Plasmodium falciparum, Plasmodium malariae, Plasmodium ovale, Plasmodium vivax, Rickettsia rickettsii, Trypanosoma brucei, and Trypanosoma cruzi. An additional 27 bacterial and viral pathogens that can be present in the blood were similarly spiked into WB. Except for the organisms targeted by the assays, the 42 pathogens were appropriately negative with both the AP/EC and BM assays, demonstrating lack of cross-reactivity (Table 4).

**TABLE 4:** Analytical specificity panel

Organism	Source	Concn
Acinetobacter baumannii complex, multiple drug resistant (MDR)	AMC^*a*^	10^6^ CFU/rx
*Aeromonas* species	AMC	10^6^ CFU/rx
Anaplasma phagocytophilum DNA	NYSDOH	10^6^ genome copies/rx
Babesia microti DNA	NYSDOH	10^6^ genome copies/rx
*Babesia odocoilei* DNA	NYSDOH	10^6^ genome copies/rx
Bacteroides fragilis DNA	ATCC 25285	10^6^ CFU/rx
Bartonella henselae DNA	NYSDOH	10^6^ genome copies/rx
Borrelia burgdorferi *B31* DNA	NYSDOH	10^6^ genome copies/rx
Citrobacter freundii	AMC	10^6^ CFU/rx
Clostridium perfringens	ATCC 13124	10^6^ CFU/rx
Ehrlichia chaffeensis DNA	NYSDOH	10^6^ genome copies/rx
Enterobacter cloacae, AmpC producing	AMC	10^6^ CFU/rx
Enterobacter cloacae, *bla*_KPC-3_	AMC	10^6^ CFU/rx
Enterococcus faecalis	AMC	10^6^ CFU/rx
Enterococcus faecium, vancomycin resistant	AMC	10^6^ CFU/rx
Escherichia coli	ATCC 25922	10^6^ CFU/rx
Escherichia coli, extended spectrum β-lactamase producing (ESB)	AMC	10^6^ CFU/rx
Hepatitis C Virus	AMC	10^6^ genome copies/rx
Human Immunodeficiency Virus	AMC	10^6^ genome copies/rx
Klebsiella pneumonia	ATCC 700603	10^6^ CFU/rx
Klebsiella pneumoniae, ESBL	AMC	10^6^ CFU/rx
Leishmania amazonensis DNA	NYSDOH	10^6^ genome copies/rx
*Leishmania guyanensis* DNA	NYSDOH	10^6^ genome copies/rx
Morganella morganii DNA	AMC	10^6^ CFU/rx
Plasmodium falciparum DNA	NYSDOH	10^6^ genome copies/rx
Plasmodium malariae DNA	NYSDOH	10^6^ genome copies/rx
Plasmodium ovale DNA	NYSDOH	10^6^ genome copies/rx
Plasmodium vivax DNA	NYSDOH	10^6^ genome copies/rx
Proteus mirabilis DNA	AMC	10^6^ CFU/rx
Pseudomonas aeruginosa, MDR	AMC	10^6^ CFU/rx
Rickettsia rickettsii DNA	NYSDOH	10^6^ genome copies/rx
Salmonella group D	AMC	10^6^ CFU/rx
Serratia marcescens	AMC	10^6^ CFU/rx
Shigella flexneri	AMC	10^6^ CFU/rx
Staphylococcus aureus, methicillin resistant	AMC	10^6^ CFU/rx
Staphylococcus aureus, methicillin susceptible	AMC	10^6^ CFU/rx
Staphylococcus epidermidis	AMC	10^6^ CFU/rx
Streptococcus agalactiae	AMC	10^6^ CFU/rx
Trypanosoma brucei DNA	NYSDOH	10^6^ genome copies/rx
Trypanosoma cruzi DNA	NYSDOH	10^6^ genome copies/rx
Yersinia enterocolitica	AMC	10^6^ CFU/rx

a Clinical specimens or isolates from Albany Medical Center.

To evaluate the performance of AP/EC on Fusion with clinical specimens, 80 whole blood samples, which were previously tested with our AP/EC TOR, were analyzed. This sample set included 50 specimens previously shown to be positive for AP. Since there were no clinical specimens positive for EC available, 30 specimens (including 20 AP positive ones) were spiked with EC plasmid DNA at concentrations of 2 or 4 log copies/reaction. To evaluate the performance of the BM PCR on Fusion with clinical specimens, 75 whole blood samples, which were previously tested with our BM TOR were analyzed. This sample set included 16 specimens previously shown to be positive for BM with our BM TOR and 59 negative specimens, of which 29 were spiked with BM plasmid DNA at various concentrations between 1.8 and 7.4 log copies/reaction. Among the 80 clinical whole blood specimens tested with Fusion AP/EC, AP was detected in 49 out of 50 positive specimens (Table 5). The one false-negative sample was retested by both the Fusion and TOR methods and was now positive by both, albeit with high Ct values (35.6 on Fusion and 34.8 by TOR). EC was detected on Fusion in all 30 spiked specimens. Similarly, among the 75 specimens tested with the BM PCR, BM was detected on the Fusion in all 16 positive and 29 spiked specimens.

**TABLE 5:** Performance with clinical specimens

Target	Result	TOR^*a*^ pos	TOR neg	Overall agreement	PPA^*b*^ (95% CI)	NPA^*c*^ (95% CI)
AP	Fusion Pos	49	0	98.8% (93.3%–99.8%)	98.0% (89.5%–99.6%)	100% (88.7%–100.0%)
	Fusion Neg	1	30			
BM	Fusion Pos	45	0	100% (95.1%–100.0%)	100.0% (92.1%–100.0%)	100% (88.7%–100.0%)
	Fusion Neg	0	30			
EC	Fusion Pos	30	0	100% (95.4%–100.0%)	100% (88.7%–100.0%)	100% (92.9%–100.0%)
	Fusion Neg	0	50			

a Test of record (TOR).

b Positive Percent Agreement (PPA).

c Negative Percent Agreement (NPA).

## DISCUSSION

**FIG 3:**
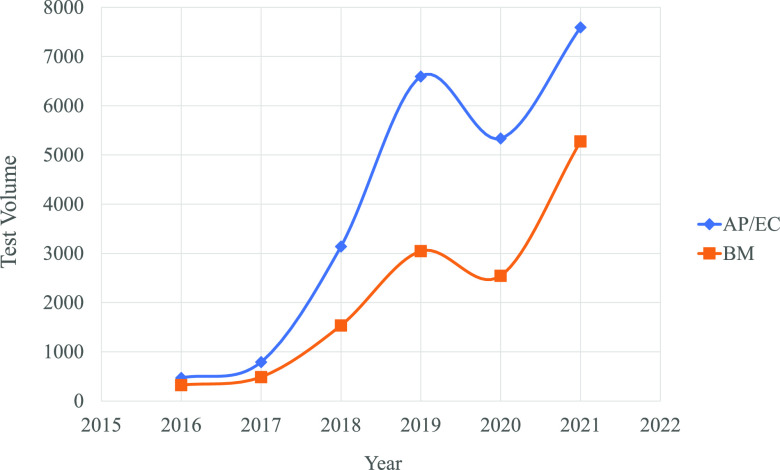
Tickborne disease test volumes. 5-year history of annual test volumes for AP/EC and BM PCR at AMC.

Tick-borne diseases are a concern in the United States with the continued increase in cases of Lyme disease, HGA, and babesiosis (15; https://www.cdc.gov/ticks/data-summary/index.html). New York State has become a hyperarea of endemicity for I. scapularis, and the bacterial, protozoal, and viral pathogens it transmits (1, 2, 15; https://www.health.ny.gov/statistics/diseases/communicable/). Since our lab began PCR testing for the detection of AP, BM and EC 5 years ago, test volumes have increased dramatically. The reason for this increase is multifaceted, but primarily due to the increased spread of infected ticks and subsequent disease in the region, but also may be to heightened physician awareness of these diseases and awareness that our lab performs this testing. In fact, by year 4, the volume of testing has increased 13- and 8-fold for AP/EC and BM testing, respectively (Fig. 3) and during the spring and summer months, our lab’s tick-borne disease NAAT testing volume is now higher than that for sexually transmitted diseases. The lower volumes seen last year were most certainly due to the effect the SARS-CoV-2 pandemic had on other areas of health care. Indeed, our current year test volumes are on track for a 22% increase for AP/EC and an 84% increase for BM over our 2019 test volumes. Hence, automation of this testing became a necessity. The Panther Fusion with the Open Access capability allows for the development of automated LDTs and provided a solution for our needs. We estimate that by implementing this system for tickborne diseases NAAT we reduce our labor needs by approximately half an FTE, based on our current test volumes.

We successfully modified two tickborne disease LDTs, AP/EC and BM, for use on the Fusion. Both LDTs on the Fusion performed similarly to our manual TORs, including a high level of agreement with clinical specimens. Actually, it was surprising that Fusion didn’t enhance the sensitivity of the LDTs. Our previous experience with a Fusion LDT for influenza A subtyping, demonstrated the system provides superior sensitivity for the detection of the virus compared to many commercial assays (16). Other studies have also shown enhanced sensitivity with Fusion (17, 18). However, the design of this study, with sample selection based on positivity and the use of spiked samples, introduced biases that would reduce the ability to observe enhanced sensitivity if it existed.

A significant limitation of this study is that we only had plasmid DNA to evaluate the performance of the EC target. Although there is precedent for the use of plasmid DNA for the evaluation of tickborne disease assay performance (13, 14), whole blood spiked plasmid DNA is not truly representative of a sample from an infected individual where the organisms are intracellular. Hence, extraction spiked DNA could be more efficient than extraction of intracellular DNA. But on the other hand, small molecules of DNA often extracted less efficiently than genomic DNA (19). The use of plasmid DNA also does not reflect gene copy number and indeed the AP genome contains more than 100 copies of msp2 (20).

It is important to recognize that there are other species of *Ehrlichia*, including *E. canis*, *E. chaffeensis*, *E. ewingii*, *E. muris*, and *E. muris eauclairensis*, which are not detected by our multiplex AP/EC. This could be problematic in regions where these organisms are endemic such as southeastern and south-central United States. Similarly, the BM target is species specific and would not detect the primary causes of babesiosis in Europe, *B. divergens* and *B. bovis*.

Rather than multiplex all the tickborne disease detection, we opted for a modular approach based on ordering practices in our area. Although HGA/HME and babesiosis can initially present similarly, physicians feel that by the time patients seek medical care, the syndromes can be differentiated based on signs and symptoms. However, given the high rates of pathogen coinfection in ticks in our area, some would argue many human infections are being missed (21). Indeed, between 2017 and the first half of 2022, 13,757 of 26,764 samples tested for AP/EC were also tested for BM. Among the population tested for all three pathogens, 7.9% were positive for AP, 2.2% were positive for BM, and 0.09% were coinfected, which demonstrates 4.3% of BM positive cases being coinfected with AP. A clinical study of signs and symptoms, ordering practices, and true infection rates would be telling.

We chose to multiplex AP and EC rather than offer individual PCRs because the disease syndromes are much more similar, and many physicians believe testing of both organisms is warranted. Furthermore, since AP was considered to be an *Ehrlichia* species until 2000, some health care providers may mistakenly still order tests for EC instead of AP (22). At this time, the incidence of EC in our area would argue against this approach. But the EC rates are increasing in NYS and there is concern that the need to test for both organisms will occur sooner than the time it takes to shift physician ordering practices.

Besides the full automation, the Fusion has numerous other benefits. For example, multiple PCRs can be run from the same nucleic acid extract. The system has on-demand testing capabilities and the ability to run LDTs alongside IVD tests, eliminating the need for batch testing or for separate instruments for LDTs and IVDs. Furthermore, we have demonstrated the PPR are stable on-board for at least 27 days and enzyme cartridges are stable for up to 60 days; however, refrigeration is needed for long-term enzyme cartridge storage. The current specimen processing workflow of whole blood, with proteinase K digestion, is a disadvantage of the system, as is the inability to add PPR mix while the system is performing any other testing. This is problematic for a system with a very broad test menu. At our facility, our instruments are in operation 24/7; hence, we have to pause testing just to add amplification mixes.

In conclusion, AP/EC and BM PCRs were successfully developed and optimized on the Panther Fusion with performance characteristics comparable to our TOR. These assays complement each other well and allow for a modular diagnostic approach for tickborne diseases, which have differing clinical presentation. Furthermore, automation of these assays will help the lab meet the demand for increased testing.

## MATERIALS AND METHODS

### Specimens.

Clinical specimens were comprised of EDTA anticoagulated whole blood (WB) including 80 received into the clinical laboratory for AP & EC and 75 for BM detection by PCR. Specimens were stored at −80°C after initial testing.

### Test of record (TOR).

Primers and probes for the AP/EC and BM real-time PCRs, designed by scientists at the Wadsworth Center, New York State Department of Health (13, 14), are detailed in Table 1 (Integrated DNA Technologies, Inc. Coralville, IA). Modification of these assays included the extraction of 0.2 mL of whole blood on a MagNA Pure 24 using MagNA Pure LC Total Nucleic Acid Isolation Kits (Roche Molecular Systems, Inc., Pleasanton, CA) with elution in 0.1 mL of buffer. Internal control DNA, EP-Bicoid and BBIC plasmid DNAs (New York State Wadsworth Laboratories, [13, 14]), were added during the extraction. Amplification for both assays were performed on SmartCyclers (Cepheid, Carlsbad, CA) with 5 μL of eluate using Perfecta Multiplex Tough Mix (Quantabio, Beverly, MA) and amplification profiles of 1 cycle of 3 min at 95°C and 45 cycles of 95°C for 15 s with 60°C for 60 s. The effective volume of whole blood analyzed was 10 μL.

### Panther Fusion LDTs.

The AP/EC and BM assays were adapted to be run on the fully automated Panther Fusion system (Fusion) with continuous, random access. Initially, 400 μL WB were diluted into 800 μL Working Diluent-Blood Transport Medium (BTM, Hologic, San Diego, CA) and 60 μL of Proteinase K (20 mg/mL) and incubated for 30 min at room temperature (15°C to 30°C) in Aptima Specimen Aliquot Tubes with penetrable caps. Automated nucleic acid extraction was performed on 300 μL of processed WB using Panther Fusion Extraction Reagent-S containing Hologic’s IC DNA. Extracted total nucleic acid was eluted in 50 μL, of which 5 μL are amplified, resulting in an effective volume of 9.5 μL whole blood analyzed. The AP/EC and BM primers and probe sequences were the same as those used with the TOR, but concentrations were optimized for use on Fusion (Table 1), as were the concentration of the Fusion Reagents (Tris [pH 8.0], KCl, MgCl_2_, IC primers and probe [Hologic]). User-prepared primer probe reconstitution (PPR) mix, with optimized reagent concentrations (Table 2) were overlaid with Panther Fusion oil reagent (Hologic) and added to the system. Fusion rehydrates the single-reaction, lyophilized enzyme and nucleotide mixture with the PPR and combines 20 μL of this master mix with eluate. The amplification profile for both Fusion LDTs was 1 cycle of 2 min at 95°C and 45 cycles of 95°C for 5 s with 60°C for 22 s.

### Plasmid DNA.

Plasmids constructed from PCR products containing AP, EC and BM target regions were obtained from Kimberlee Musser, Wadsworth Center, as were the EP-Bicoid and BBIC plasmids. The EP-Bicoid and BBIC plasmids were designed to be used as internal control DNA with heterologous DNA flanked by the primer binding sites for the EC *dsb* and BM 18S rRNA genes, respectively. Target specific plasmids were serially diluted 1:4 or 1:10 in molecular grade water, and the dilutions added to WB for testing 4 to 10 times for LOD, linearity and precision analyses. Target specific plasmid dilutions in WB were also used for optimization studies, stability studies, control material and spiking into clinical specimens to provide additional positive samples for EC and BM. Positive controls were comprised of plasmid DNA spiked into BTM treated WB to concentrations of 43 copies/reaction (c/rx) of AP, 500 c/rx of BM and 49 c/rx of EC.

### Statistical analysis.

Specimens were defined as true positive (TP) if they were positive based on TOR testing. Positive and negative predictive values (PPV and NPV) with confidence intervals (CI, Clopper-Pearson Method) (23) and linear regressions were determined using Microsoft Excel 2016 (Redmond, WA). Probit analyses for the LOD with a 95% probability of detection were performed using SPSS version 13.0 (IBM, Armonk, NY).
